# Supplementary material for: Saccharomyces boulardii (CNCM I-745) improves intestinal damage in sepsis by remodeling intestinal flora structure
Source: Front Cell Infect Microbiol. 2025 Jul 31;15:1602792. doi: 10.3389/fcimb.2025.1602792 (PMC12350329; doi:10.3389/fcimb.2025.1602792)
Supplement: Supplementary file 1 [file DataSheet1.zip › summary/summary/6_Taxonomy_community/1_Taxonomy_profiling/4_Order/Group/Order_abund_top30.Group.cluster.pdf]

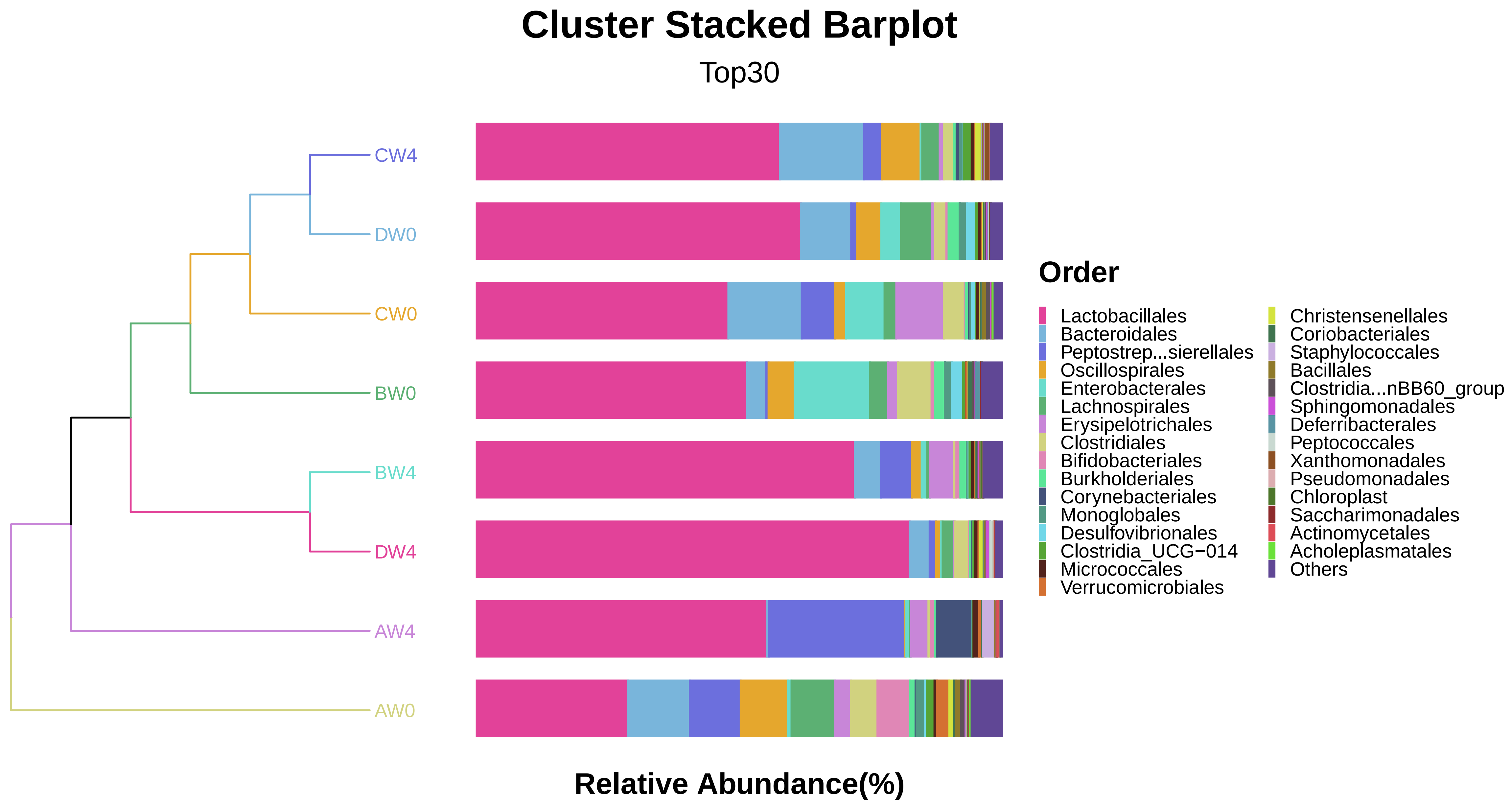
Cluster Stacked Barplot
Top30
CW4
DW0
CW0
BW0
BW4
DW4
AW4
AW0
Relative Abundance(%)
Order
Lactobacillales
Bacteroidales
Peptostrep...sierellales
Oscillospirales
Enterobacterales
Lachnospirales
Erysipelotrichales
Clostridiales
Bifidobacteriales
Burkholderiales
Corynebacteriales
Monoglobales
Desulfovibrionales
Clostridia_UCG−014
Micrococcales
Verrucomicrobiales
Christensenellales
Coriobacteriales
Staphylococcales
Bacillales
Clostridia...nBB60_group
Sphingomonadales
Deferribacterales
Peptococcales
Xanthomonadales
Pseudomonadales
Chloroplast
Saccharimonadales
Actinomycetales
Acholeplasmatales
Others
